# Effect of magnetic field, heat generation and absorption on nanofluid flow over a nonlinear stretching sheet

**DOI:** 10.3762/bjnano.11.82

**Published:** 2020-07-02

**Authors:** Santoshi Misra, Govardhan Kamatam

**Affiliations:** 1 Department of Mathematics, St. Ann’s College for Women, Mehdipatnam, Hyderabad, Telangana, India; 2 Department of Mathematics, GITAM University, Hyderabad, India

**Keywords:** Brownian motion, heat generation and absorption, magnetic field, nanofluid, thermophoresis

## Abstract

The study of magnetohydrodynamic flow of a nanoparticle suspension under the influence of varied dimensionless parameters has been the focus of research in contemporary times. This work models the effect of magnetic field, heat generation and absorption parameter in a steady, laminar, two-dimensional boundary layer flow of a nanofluid over a permeable stretching sheet at a given surface temperature and partial slip. The highly nonlinear governing equations are solved numerically using similarity transformations with suitable boundary conditions and converted to ordinary differential equations. A computational model is setup using FORTRAN, where a relevant Adam’s predictor–corrector method is employed to solve the equations. The impact of the dimensionless parameters, including the Brownian motion, thermophoresis, magnetic field, heat generation and absorption parameters, on the velocity, temperature and nanoparticle concentration of fluid flow are analysed systematically.

## Introduction

The study of magnetohydrodynamic problems, such as nanofluid flow over a permeable stretching sheet, has recently become relevant due to potential applications in various fields of science, such as metallurgy and chemical engineering processes with industrial applications which include glass fibre, paper production, hot rolling, metal spinning, wire drawing, etc. Research involving two dimensional boundary layer laminar flow was initiated by Sakiadis [1], who later on extended these studies in order to include continuous surfaces and the boundary layer behaviour [2]. This research was continued by Crane [3], who considered the Navier–Stokes equations involving the conservation of mass, momentum, energy and concentration for the boundary layer flow. Other groups, such as Chamka et al. [4], studied the fluid flow using a semi-infinite flat surface with the heat generation and absorption coefficient. Anderson [5] conducted experiments on fluid flow using the finite difference method which seemed amenable to provide accurate results. The problem involving laminar flow due to stretching of the sheet in nanofluids was investigated by Khan and Pop [6] which gained enormous popularity among researchers.

The study of nanofluids (i.e., a fluid containing particles smaller than 100 nm in at least one dimension) is important mainly due to the fact that they can be used to enhance the thermal conductivity and convective heat transfer performance of base fluids such as water, ethylene, glycol, etc. This takes place due to the intense and rigorous distribution of nanoparticle Brownian motion within the base fluid, thus enhancing the uniformity, conductance and properties which have paved the way for substantial research in this field.

Sheet stretching involves analysis using both linear and nonlinear equations (e.g., the polymer extrusion process) as reflected in the numerical study by Rana and Bhargava [7]. This study was extended by Das [8] and Hayat et al. [9], who used partial slip conditions for the boundary layer flow to investigate the velocity, temperature and concentration changes with regard to various dimensionless parameters in the fluid flow under the influence of a magnetic field. Besthapu and Bandari [10] have analysed the heat and mass transfer rates using Casson nanofluids with nonlinear equations. Applications in nuclear waste storage were initiated by Gaffar et al. [11], who focussed on viscoelastic Jeffrey fluid in a porous medium considering the effect of various dimensionless parameters that influence the heat and mass transfer flow. Dogonchi and Ganji [12] examined the velocity and temperature of nanofluids under the thermal radiation effect using Brownian motion. Viscous fluid flow melting following plate thickness variation was systematically investigated by Farooq et al. [13]. Qayyum et al. [14] considered external factors acting upon the fluid focussing on the influence of heat generation/absorption phenomena in the flow problem. Sreekala et al. [15], Rashid et al. [16] and Ahmad et al. [17] included external forces acting on the fluid flow, emphasizing their tremendous effect on the velocity, temperature and concentration profiles with fluid heat and mass transfer. Seth et al. [18] and Soomro et al. [19–20] have extended nanofluid research by considering the effects of various dimensionless parameters on the nanoparticle flow when suspended in different nanofluids. Farooq et al. [21], Irfan et al. [22] and Pal et al. [23] have supplemented the investigation by extending it to the three dimensional flow of different nanofluids which were used to obtain the dimensionless velocity, temperature and concentration profiles under the influence of external fields. In order to study nanofluid heat and mass transfer flow, Shah et al. [24] and Yousif et al. [25] developed mathematical models to obtain the numerical solution of basic single-variable governing equations.

Keeping in mind the prior investigations involving steady boundary layer flow and heat transfer of a nanofluid through a permeable stretching surface in the presence of partial slip, the present research incorporates the magnetic field effect and heat generation/absorption coefficient into the velocity, temperature and concentration profiles. Given that the end product is proportional to the heat transfer rate, the most frequent topic of boundary layer flow is the heat exchange phenomenon which has applicability to real life problems. Here, the elucidation of extrinsic forces acting on the flow with regard to partial differential equations has been reduced to ordinary differential equations with distinct boundary conditions, and the results were interpreted graphically and are corroborated by the literature.

## Methods

### Problem motivation, governing equations and problem solution

**Figure 1:**
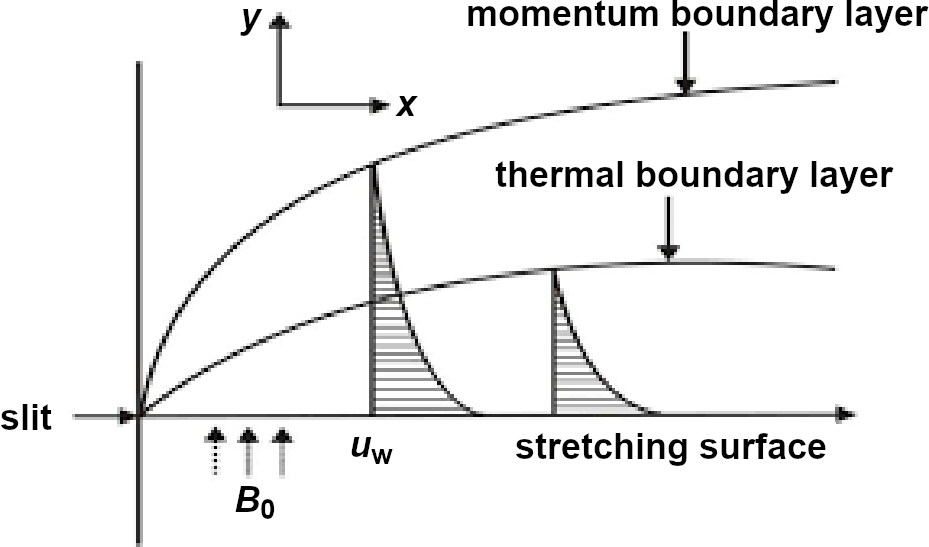
Two-dimensional coordinate system and fluid flow mechanism of a nanoparticle suspension.

A two-dimensional, laminar, nonlinear steady-state boundary layer flow of a nanofluid across a permeable stretching surface was considered for study, as shown in Figure 1.

Figure 1 describes the stretching surface of a sheet taken along the *x* and *y* axes, normal to the surface. Fluid flow occurs when *y* ≥ 0, which is triggered when the sheet is stretched out of the slit occurring at the origin (when *x* = *y* = 0). The flow speed at any arbitrary point on the plate is assumed to be proportional to the power of its distance from the slit, considering boundary layer flow approximations at a distance of *x* units from the farthest edge. Considering a fixed origin, the sheet velocity is represented by *u*_w_(*x*) = *ax^n^*, where *n* is the stretching parameter, *a* is the constant and *x* is the coordinate along the stretching surface.

The basic nanofluid governing equations in Cartesian form are given in the following where *T*_w_ and *C*_w_ represent the sheet temperature and the sheet nanoparticle fraction, respectively. The pressure and external forces are neglected and the nanoparticle fraction is assumed to be constant across the stretching surface. *T*_∞_ and *C*_∞_ denote ambient temperature and the associated nanoparticle fraction where *T*_w_ > *T*_∞_ and *C*_w_ > *C*_∞_. This physical model has significant applications in modern nanotechnology and thermal manufacturing in various industries.

#### Conservation of mass equation

[1] $$\frac{\text{d}u}{\text{d}x}+\frac{\text{d}v}{\text{d}y}=0$$

#### Conservation of momentum equation (velocity)

[2] $$u\frac{\text{d}u}{\text{d}x}+v\frac{\text{d}v}{\text{d}y}=v\frac{{\text{d}}^{2}u}{\text{d}y^{2}}-\frac{\sigma B_{0}^{2}}{\rho_{\text{f}}}u$$

#### Conservation of thermal energy equation (temperature)

[3] $$\begin{array}{c} u\frac{\partial T}{\partial x}+v\frac{\partial T}{\partial y}=\alpha_{\text{m}}\nabla^{2}T+\tau \left[D_{\text{B}}\frac{\partial C}{\partial y}\frac{\partial T}{\partial y}+\left(\frac{D_{\text{T}}}{T_{\infty }}\right){\left(\frac{\partial T}{\partial y}\right)}^{2}\right]\\ +\frac{1}{\rho c_{\text{p}}}Q_{0}\left(T-T_{\infty }\right) \end{array}$$

#### Conservation of nanoparticle equation (concentration)

[4] $$u\frac{\partial C}{\partial x}+v\frac{\partial C}{\partial y}=D_{\text{B}}\frac{\partial^{2}C}{\partial y^{2}}+\left(\frac{D_{\text{T}}}{T_{\infty }}\right)\frac{\partial^{2}T}{\partial y^{2}}$$

which is subjected to the following boundary conditions

[5] $$\text{At }y=0:\;\;u=u_{\text{w}}+u_{\text{s}},\;\;v=\pm v_{\text{w}},\;\;T=T_{\text{w}},\;\;C=C_{\text{w}}$$

[6] $$y\to \infty :\;\;u\to 0,\;\;T\to T_{\infty },\;\;C\to C_{\infty }$$

and

[7] $$\alpha_{\text{m}}=\frac{K_{\text{m}}}{{\left(\rho c\right)}_{\text{f}}},\;\;\tau =\frac{{\left(\rho c\right)}_{\text{p}}}{{\left(\rho c\right)}_{\text{f}}}$$

α_m_ denotes the thermal diffusivity, and τ denotes the ratio between effective heat capacities of the nanoparticle material and the base fluid. In Equations 1–4, *u*, and *v* denote the velocity components along *x* and *y* axes, respectively, ρ_f_ is the base fluid density, *a* is a positive constant, *D*_B_ is the Brownian diffusion coefficient, *D*_T_ denotes the thermodiffusion coefficient, *c* is the volumetric expansion coefficient, ρ_p_ is the particle density, σ denotes the nanofluid electrical conductivity, *B*_0_ denotes the magnetic induction, *v*_w_ denotes the suction/injection velocity and *Q*_0_ (*Q*) denotes the heat generation (absorption) coefficient.

*u*_s_ in Equation 8 represents the slip velocity, given as

[8] $$u_{\text{s}}=l\frac{\partial u}{\partial y}\text{ at }y=0$$

which is proportional to the local sheet stress, and *l* is the slip length constant.

#### The similarity transformations

The similarity transformations to solve the governing equations are as follows:

[9] $$\begin{array}{l} \eta =y\sqrt{\frac{a\left(n+1\right)}{2v}}x^{\frac{n-1}{2}},\;\;u=ax^{n}f^{\prime }\left(\eta \right),\\ v=-\sqrt{\frac{av\left(n+1\right)}{2}}x^{\frac{n-1}{2}}\left[f\left(\eta \right)+\left(\frac{n-1}{n+1}\right)\eta f^{\prime }\left(\eta \right)\right],\\ \theta \left(\eta \right)=\frac{T-T_{\infty }}{T_{\text{w}}-T_{\infty }},\;\;\phi \left(\eta \right)=\frac{C-C_{\infty }}{C_{\text{w}}-C_{\infty }} \end{array}$$

By substituting the similarity transformations in Equation 9 into the governing boundary layer Equations 1–4 they reduce to ordinary differential equations:

[10] $$f^{\prime \prime \prime }+ff^{\prime \prime }-\left[\frac{2n}{n+1}\right]{f^{\prime }}^{2}-Mf^{\prime }=0$$

[11] $$\frac{1}{\mathrm{Pr}}\theta^{\prime \prime }+f\theta^{\prime }+\text{Nb}\theta^{\prime }\phi^{\prime }+\text{Nt}{\left(\theta^{\prime }\right)}^{2}+Q\theta =0$$

[12] $$\phi^{\prime \prime }+\text{Le}f\phi^{\prime }+\frac{\text{Nt}}{\text{Nb}}\theta^{\prime \prime }=0$$

The transformed boundary conditions of Equation 5 and Equation 6, with regard to the similarity transformations in Equation 9, are:

[13] $$\text{At }\eta =0:\;\;f=F_{\text{w}},\;\;f^{\prime }=1+\xi_{\text{p}}f^{\prime \prime },\;\;\theta =1,\;\;f=1$$

[14] $$\eta \to \infty :\;\;f^{\prime }\to 0,\;\;\theta \to 0,\;\;\phi \to 0$$

The working rules for the boundary conditions are:

At *y* = 0: *v* = ±*v*_w_, where


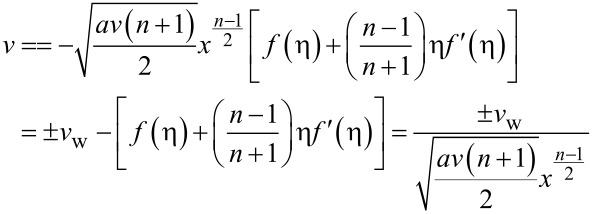


at η = 0:


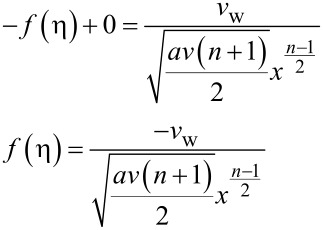


Therefore, *f*(η) = *F*_w_ where


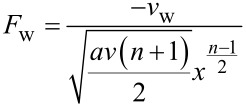


at *y* = 0: *u* = *u*_w_ + *u*_s_ where *u*_w_ = *ax^n^*, 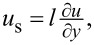 *u* = *ax^n^f*´(η) where


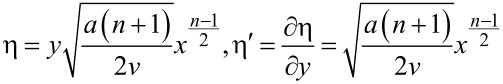


substituting the above values into *u* = *u*_w_ + *u*_s_ we get:


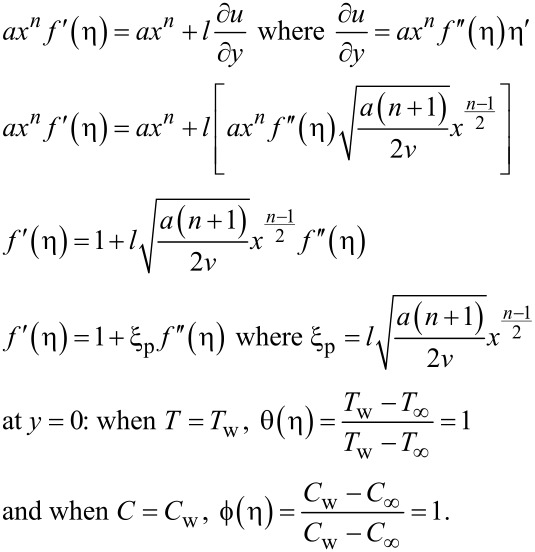


Therefore, at η = 0: *f* = *F*_w_, *f*´ = 1 + ξ_p_*f*´´, θ = 1, ϕ = 1 when *y* → ∞: *u* → 0, *T* → *T*_∞_, *C* → *C*_∞_ henceforth as *u* → 0, *f*´(η) → 0, as *T* → *T*_∞_, θ(η) → 0, as *C* → *C*_∞_, ϕ(η) → 0

Therefore, at η → ∞: *f*´ → 0, θ → 0, ϕ → 0

The prime (´) denotes differentiation with respect to η, and the physical parameters involved in the equations are defined as follows:

[15] $$\begin{array}{l} \mathrm{Pr}=\frac{v}{\alpha_{\text{m}}}\text{ Prandtl number, Le}=\frac{v}{D_{\text{B}}}\text{ Lewis number,}\\ \text{Nb}=\frac{{\left(\rho c\right)}_{\text{p}}D_{\text{B}}\left(C_{\text{w}}-C_{\infty }\right)}{{\left(\rho c\right)}_{\text{f}}v}\text{ Brownian motion parameter,}\\ \text{Nt}=\frac{{\left(\rho c\right)}_{\text{p}}D_{\text{T}}\left(T_{\text{w}}-T_{\infty }\right)}{{\left(\rho c\right)}_{\text{f}}vT_{\infty }}\text{ Thermophoresis parameter,}\\ F_{\text{w}}=\frac{-v_{\text{w}}}{\sqrt{\frac{av\left(n+1\right)}{2}}x^{\frac{n-1}{2}}}\text{ Suction/injection parameter,}\\ \xi_{\text{p}}=l\sqrt{\frac{a\left(n+1\right)}{2}}x^{\frac{n-1}{2}}\text{ Slip parameter for liquids,}\\ M=\frac{\sigma B_{0}^{2}}{\rho_{\text{f}}}\frac{2}{a\left(n+1\right)x^{n-1}}\text{ Magnetic parameter,}\\ Q=\frac{2Q_{0}}{\rho c_{\text{p}}a\left(n+1\right)x^{n-1}}\text{ Heat generation/absorption coefficient} \end{array}$$

The abbreviations used for the calculations are summarized in Table 1.

**Table 1:** Nomenclature for the basic nanofluid governing equations.

Roman		Greek symbols	

*a*	constant	α_m_	thermal diffusivity
*n*	nonlinear stretching parameter	ξ_p_	slip parameter for liquids
*C*	nanoparticle volume fraction	υ	kinematic viscosity of the fluid
*C*_w_	nanoparticle volume fraction at the sheet	τ	torsion parameter defined by 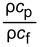
*C*_∞_	ambient nanoparticle volume fraction	ρ*c*_p_	effective heat capacity of nanoparticles
Nt	thermophoresis parameter	ρ*c*_f_	heat capacity of the fluid
Nb	Brownian motion parameter	η	similarity V
(*x*,*y*)	Cartesian coordinates	ϕ(η)	rescaled nanoparticle volume fraction
*T*_w_	temperature at the sheet	θ(η)	dimensionless temperature
*T*_∞_	ambient temperature	ρ_f_	base fluid density
*T*	temperature	ρ_p_	nanoparticle mass density
*u*_s_	slip velocity		
*v*_w_	suction/injection		
*D*_B_	Brownian diffusion coefficient		
*D*_T_	thermodiffusion coefficient		
*u*_w_	velocity of the stretching sheet		
*M*	magnetic field parameter		
*Q*_0_*/Q*	heat generation/absorption coefficient		
Le	Lewis number		
*F*_w_	suction/injection parameter		
*u*, *v*	velocity components along *x*, *y* axes		
σ	nanofluid electrical conductivity		
*B*_0_	magnetic induction		
*l*	slip length constant		
Pr	Prandtl number		

#### Solving a system of first-order differential equations

The nonlinear and coupled partial differential equations, represented by Equations 10–12, are solved by using the Adam’s predictor–corrector method which is the most efficient technique in numerical analysis used to solve distinctive problems related to heat transfer, fluid mechanics, and electrical systems. The first step involves reducing the nonlinear differential equations of third order in *f* and second order in θ and ϕ to a system of first-order differential equations, in order to simplify the problem as follows.

[16] $$f^{\prime \prime \prime }=-ff^{\prime \prime }+\frac{2n}{n+1}{f^{\prime }}^{2}+Mf^{\prime }$$

[17] $$\theta^{\prime \prime }=-\mathrm{Pr}\left[f\theta^{\prime }+\text{Nb}\theta^{\prime }\phi^{\prime }+\text{Nt}{\left(\theta \right)}^{2}+Q\theta \right]$$

[18] $$\phi^{\prime \prime }=-\text{Le}f\phi^{\prime }-\frac{\text{Nt}}{\text{Nb}}\theta^{\prime \prime }$$

These equations were solved independently using a FORTRAN program which converts each one of them using different variables to first order as follows.

Equation 16 is represented as a system of equations for *f* as:


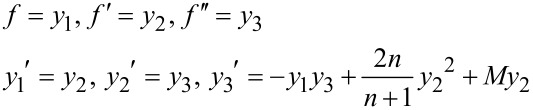


The boundary conditions with respect to η for *f* are:


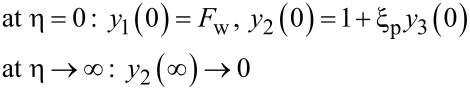


We assume an initial guess value as α for *y*_3_, i.e., *y*_3_(0) = α. Hence, *y*_1_(0) = *F*_w_, *y*_2_(0) = 1 + ξ_p_α, *y*_3_(0) = α where α needs to be obtained by using Newton’s method to satisfy *y*_2_(η_∞_) → 0.

Equation 17 and Equation 18 are represented as follows by defining the system of equations for θ and ϕ:


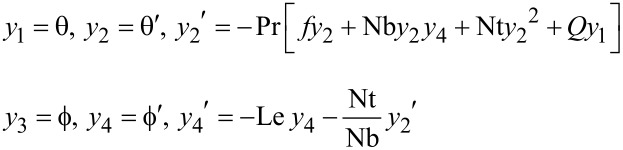


The boundary conditions with respect to η for θ and ϕ are as follows:


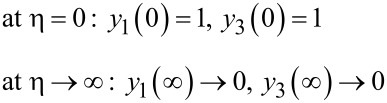


The values of α and β, which are the unknowns with respect to θ and ϕ, are obtained by starting with an initial guess and then correcting the values using Newton’s method to satisfy the end conditions as follows:


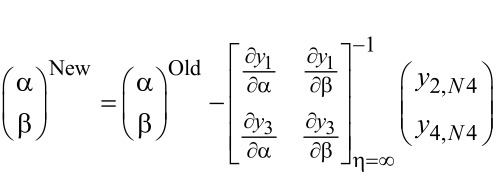


The boundary value problem is converted to an initial value problem by reducing the equations, as shown above. The initial value problem is solved by using Adam’s predictor–corrector method and assigning an approximate initial value with step size *h* = 0.01 and η = 4 at the maximum value. The solution is obtained for different parameters used and convergence is achieved with a change in a single parameter. The criteria for convergence are the approximation up to three significant digits. The results derived through computation are plotted as graphs for a clear picture of the numerical problem (Table 2, Table 3).

**Table 2:** Computation of the streamwise velocity, temperature and concentration profiles for different values of the magnetic field parameter, *M*.

η	*M* = 0.5			*M* = 1.0			*M* = 2.0			*M* = 3.0		
	*f*´(η)	θ(η)	ϕ(η)	*f*´(η)	θ(η)	ϕ(η)	*f*´(η)	θ(η)	ϕ(η)	*f*´(η)	θ(η)	ϕ(η)

0	0.468	1	1	0.428	1	1	0.375	1	1	0.339	1	1
0.2	0.373	0.935	0.714	0.328	0.94	0.726	0.268	0.946	0.74	0.23	0.949	0.749
0.4	0.298	0.855	0.51	0.252	0.866	0.528	0.193	0.881	0.549	0.156	0.889	0.563
0.6	0.238	0.766	0.374	0.193	0.784	0.392	0.138	0.808	0.415	0.106	0.823	0.429
0.8	0.19	0.672	0.286	0.149	0.699	0.302	0.099	0.732	0.323	0.072	0.753	0.335
1.0	0.152	0.581	0.229	0.114	0.614	0.242	0.071	0.656	0.259	0.049	0.681	0.269
1.2	0.122	0.494	0.189	0.088	0.532	0.2	0.051	0.581	0.214	0.033	0.611	0.221
1.4	0.098	0.414	0.159	0.068	0.456	0.17	0.037	0.51	0.18	0.023	0.543	0.186
1.6	0.078	0.342	0.136	0.052	0.386	0.145	0.027	0.443	0.155	0.015	0.478	0.159
1.8	0.062	0.279	0.115	0.04	0.323	0.125	0.019	0.38	0.134	0.01	0.417	0.137
2.0	0.049	0.225	0.097	0.031	0.267	0.107	0.014	0.323	0.115	0.007	0.359	0.119
2.2	0.039	0.179	0.08	0.023	0.218	0.09	0.01	0.272	0.099	0.005	0.306	0.102
2.4	0.031	0.14	0.066	0.018	0.175	0.075	0.007	0.225	0.084	0.003	0.257	0.087
2.6	0.024	0.107	0.053	0.014	0.138	0.062	0.005	0.183	0.071	0.002	0.213	0.074
2.8	0.018	0.081	0.041	0.01	0.107	0.049	0.004	0.146	0.058	0.001	0.172	0.061
3.0	0.014	0.059	0.031	0.007	0.08	0.038	0.003	0.113	0.046	0.001	0.135	0.049
3.2	0.01	0.042	0.022	0.005	0.058	0.028	0.002	0.084	0.035	0.001	0.102	0.038
3.4	0.007	0.027	0.015	0.004	0.039	0.02	0.001	0.058	0.025	0	0.072	0.028
3.6	0.004	0.016	0.009	0.002	0.024	0.012	0.001	0.036	0.016	0	0.045	0.018
3.8	0.002	0.007	0.004	0.001	0.011	0.006	0	0.017	0.008	0	0.021	0.009
4.0	0	0	0	0	0	0	0	0	0	0	0	0

**Table 3:** Computation of streamwise velocity, temperature and concentration profiles for different values of the heat generation/absorption coefficient, *Q*.

η	*Q* = 0.0			*Q* = 0.2			*Q* = 0.3		
	*f*´(η)	θ(η)	ϕ(η)	*f*´(η)	θ(η)	ϕ(η)	*f*´(η)	θ(η)	ϕ(η)

0	0.524	1	1	0.524	1	1	0.524	1	1
0.2	0.437	0.928	0.697	0.437	0.95	0.682	0.437	1.028	0.627
0.4	0.365	0.839	0.485	0.365	0.88	0.462	0.365	1.035	0.367
0.6	0.306	0.738	0.349	0.306	0.794	0.322	0.306	1.018	0.208
0.8	0.256	0.634	0.263	0.256	0.701	0.238	0.256	0.977	0.124
1.0	0.214	0.533	0.209	0.214	0.605	0.189	0.214	0.918	0.088
1.2	0.179	0.439	0.172	0.179	0.513	0.158	0.179	0.845	0.076
1.4	0.149	0.355	0.143	0.149	0.427	0.135	0.149	0.764	0.076
1.6	0.124	0.283	0.119	0.124	0.35	0.117	0.124	0.678	0.079
1.8	0.103	0.221	0.099	0.103	0.281	0.1	0.103	0.591	0.082
2.0	0.085	0.17	0.08	0.085	0.223	0.085	0.085	0.506	0.082
2.2	0.069	0.129	0.064	0.069	0.174	0.07	0.069	0.424	0.079
2.4	0.056	0.096	0.05	0.056	0.133	0.057	0.056	0.348	0.074
2.6	0.045	0.071	0.038	0.045	0.1	0.045	0.045	0.278	0.067
2.8	0.035	0.051	0.028	0.035	0.073	0.035	0.035	0.216	0.058
3.0	0.027	0.036	0.021	0.027	0.052	0.026	0.027	0.162	0.048
3.2	0.02	0.024	0.014	0.02	0.036	0.018	0.02	0.115	0.037
3.4	0.014	0.015	0.009	0.014	0.023	0.012	0.014	0.076	0.027
3.6	0.008	0.009	0.005	0.008	0.013	0.007	0.008	0.045	0.017
3.8	0.004	0.004	0.002	0.004	0.006	0.003	0.004	0.019	0.008
4.0	0	0	0	0	0	0	0	0	0

## Results and Discussion

To provide physical insight into the flow problem, numerical computations involving various parameters and their influence on the dimensionless velocity, temperature and nanoparticle concentration of fluid flow have been represented graphically as follows.

### Impact of ξ on *f*´(η)

**Figure 2:**
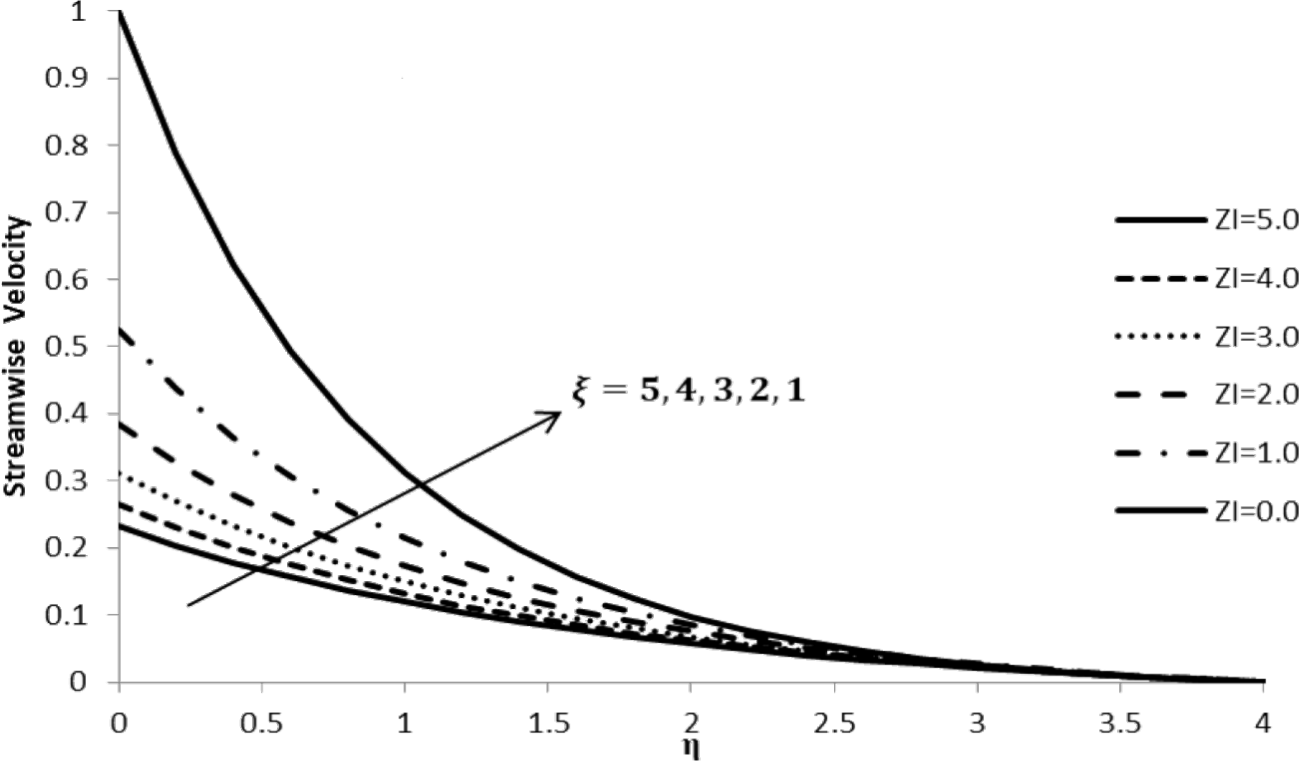
Influence of the slip parameter for liquids ξ on the streamwise velocity component *f*´(η) when *n* = 2.0, *M* = 0.0, *Q* = 0.0, Pr = 2.0, Nb = Nt = 0.5, Le = 5.0, and *F*_w_ = 0.2.

Figure 2 illustrates the influence of the slip parameter, ξ, on the velocity gradient which decreases with an increase in the value of ξ, converging to zero at the end of the boundary layer, thus causing a reduction in its thickness for nanofluids. This is due to the fact that an increase in the slip parameter causes a reduction in the skin friction at the surface acting between the stretching sheet and the fluid flow, thus drastically decreasing the velocity gradient.

### Impact of ξ on θ(η)

**Figure 3:**
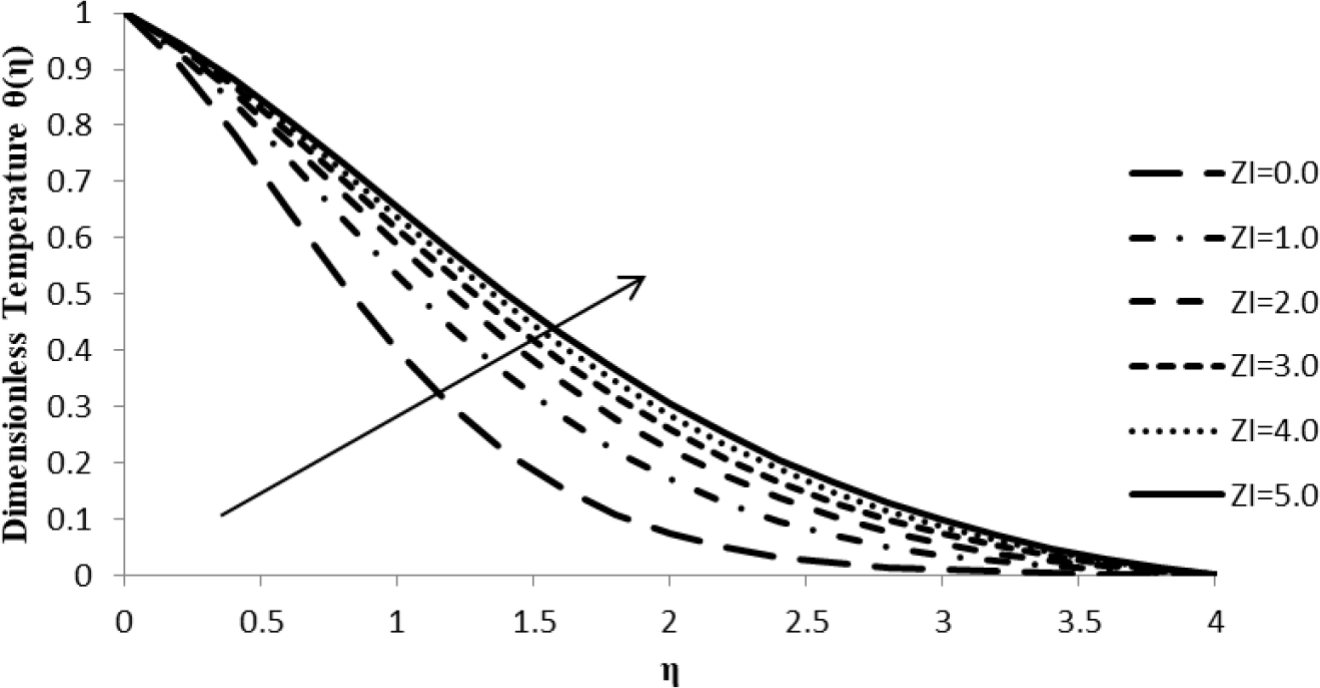
Influence of the slip parameter for liquids ξ on the temperature distribution θ(η) when *n* = 2.0, *M* = 0.0, *Q* = 0.0, Pr = 2.0, Nb = Nt = 0.5, Le = 5.0, and *F*_w_ = 0.2.

The temperature variation component, θ(η), increases with an increase in the slip parameter, ξ, which further leads to an increase in the fluid temperature, thus intensifying the thermal boundary layer thickness (Figure 3). An increase in the slip parameter causes friction at the surface which, in turn, generates a frictional force allowing more fluid to flow passed the stretching sheet, causing an increase in the temperature gradient and reducing the velocity of the fluid.

### Impact of ξ on ϕ(η)

**Figure 4:**
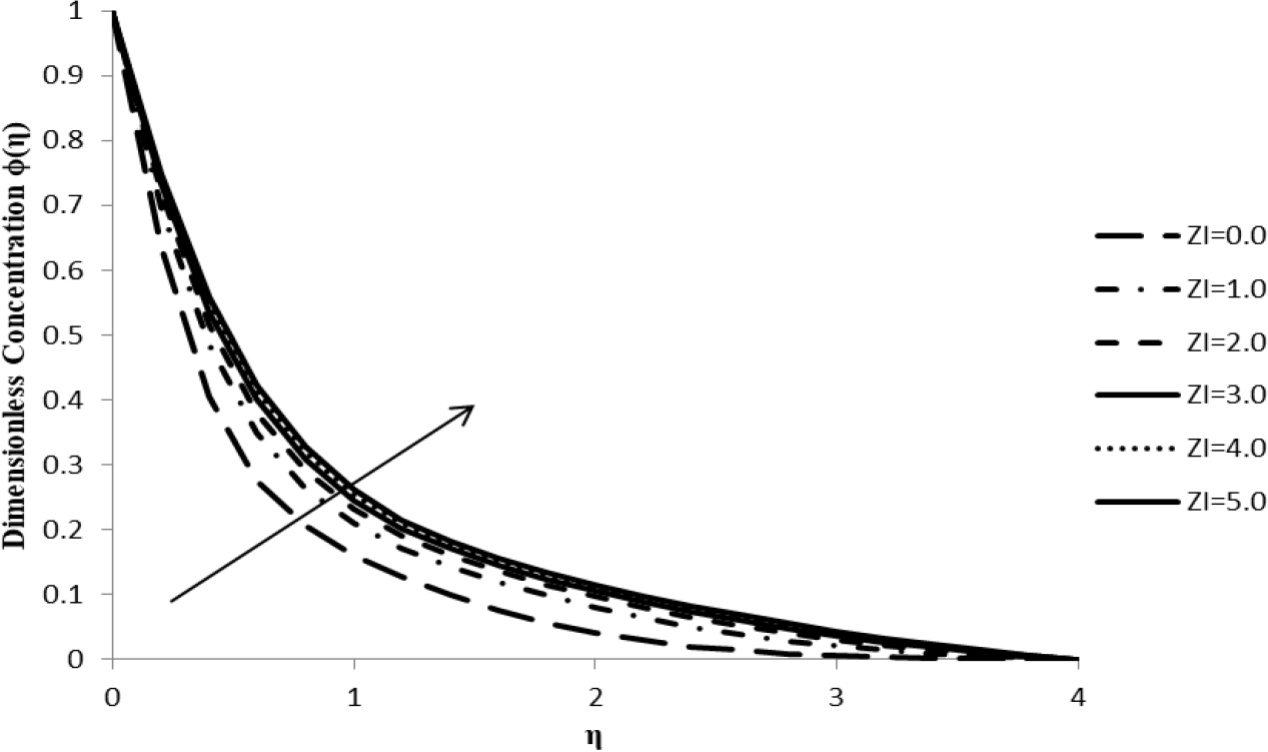
Influence of the slip parameter for liquids ξ on the concentration distribution ϕ(η) when *n* = 2.0, *M* = 0.0, *Q* = 0.0, Pr = 2.0, Nb = Nt = 0.5, Le = 5.0, and *F*_w_ = 0.2.

The nanoparticle concentration distribution, ϕ(η), increases with an increase in the slip parameter, ξ, at a given constant surface temperature. An increase in the slip parameter causes friction at the surface which, in turn, generates a frictional force allowing more fluid to flow passed the stretching sheet. This causes an increase in concentration distribution of the fluid as shown in Figure 4, which ultimately reduces the fluid velocity.

### Impact of *n* on *f*´(η)

**Figure 5:**
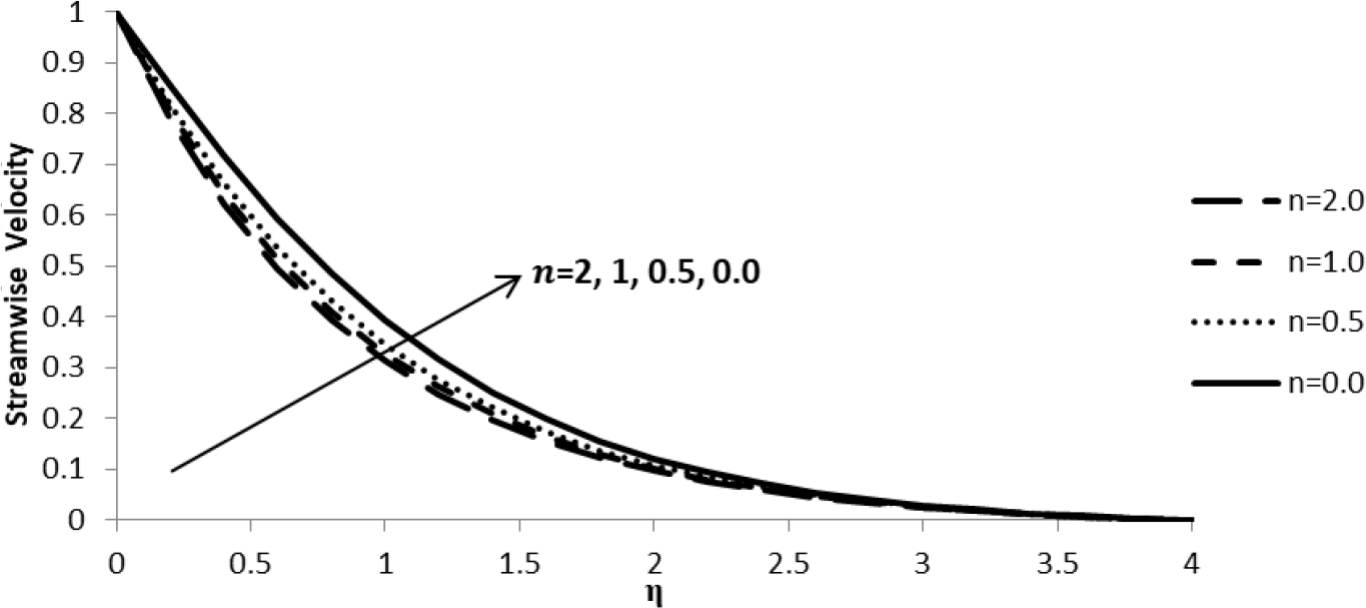
Influence of the stretching parameter *n* on the streamwise velocity component *f*´(η) when ξ = *M* = *Q* = 0.0, Pr = 2.0, Nb = Nt = 0.5, Le = 5.0, and *F*_w_ = 0.2.

The nonlinear stretching parameter, *n*, has a significant influence on the fluid flow velocity component, *f*´(η). Figure 5 shows that with an increase in *n*, the velocity gradient decreases, thus depleting the thickness of the momentum boundary layer. This is due to the fact that, with the sheet being stretched, there is a retarding force in the fluid in which the fluid particles have severe impact on the velocity component leading to its reduction as we move away from the boundary layer.

### Impact of *n* on θ(η)

**Figure 6:**
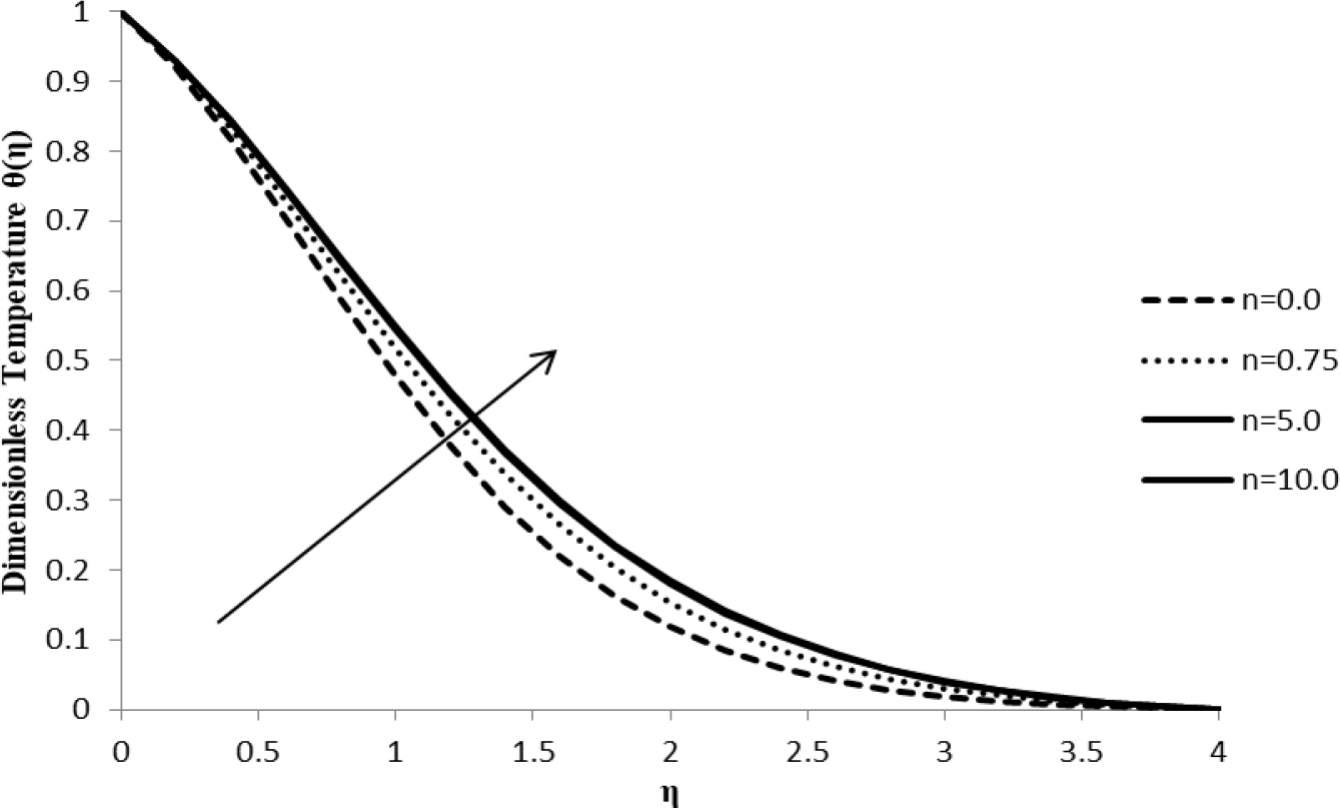
Influence of the stretching parameter *n* on the temperature distribution θ(η) when ξ = 1.0, *M* = *Q* = 0.0, Pr = 2.0, Nb = Nt = 0.5, Le = 5.0, and *F*_w_ = 0.2 (due to the boundary condition, the value of ξ is changed).

With an increase in the stretching parameter, *n*, the temperature gradient, θ(η), increases leading to an increase in the thermal boundary layer thickness. As the value of the stretching parameter increases, the convection process in the particles of the fluid flow intensifies, thus leading to a rapid increase in the temperature gradient which, in turn, is responsible for an increase in the thermal boundary layer thickness (Figure 6).

### Impact of *n* on ϕ(η)

**Figure 7:**
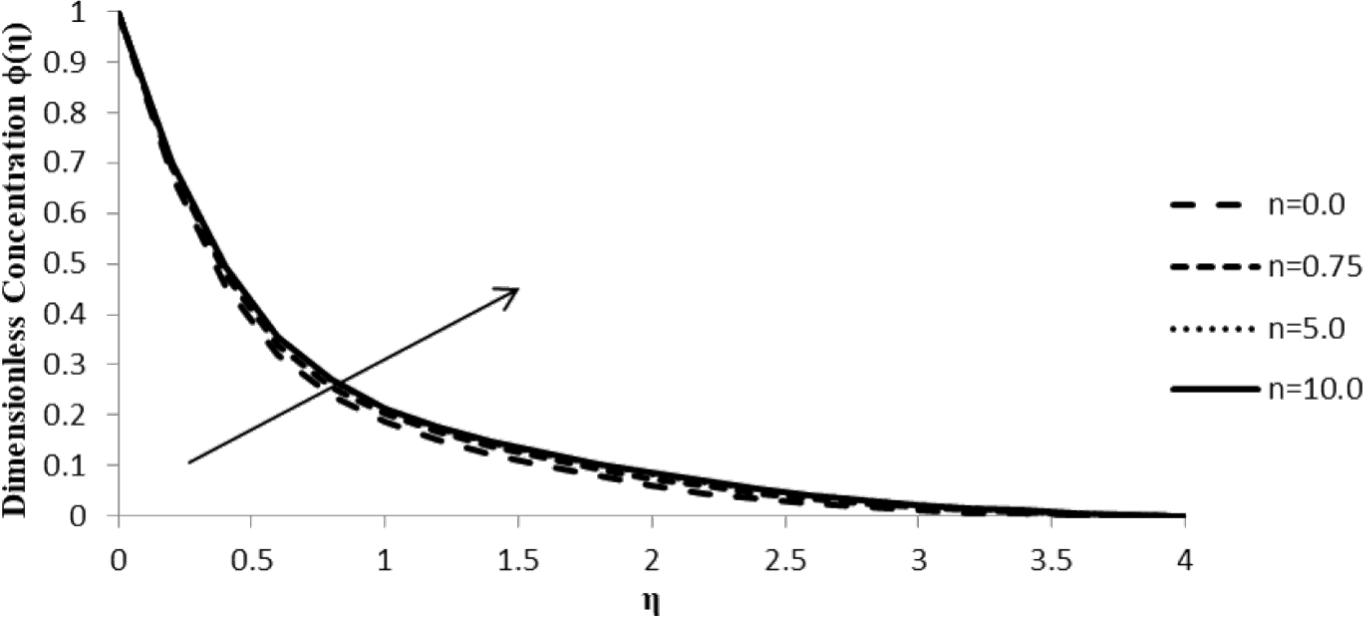
Influence of the stretching parameter *n* on the concentration distribution ϕ(η) when ξ = 1.0, *M* = *Q* = 0.0, Pr = 2.0, Nb = Nt = 0.5, Le = 5.0, and *F*_w_ = 0.2 (due to the boundary condition, the value of ξ is changed).

With an increase in the stretching parameter, *n*, the concentration profile of the fluid, ϕ(η), increases slightly but the effect observed is not significant, which is noticed at higher values of *n*. At lower values of the stretching parameter, there is a negligible change in the fluid concentration of the fluid, which is independent of stretch. On the other hand, there is a significant increase in the stretching parameter at extremely high values that does not seem to affect the fluid particle concentration (Figure 7).

### Impact of Nb on θ(η)

**Figure 8:**
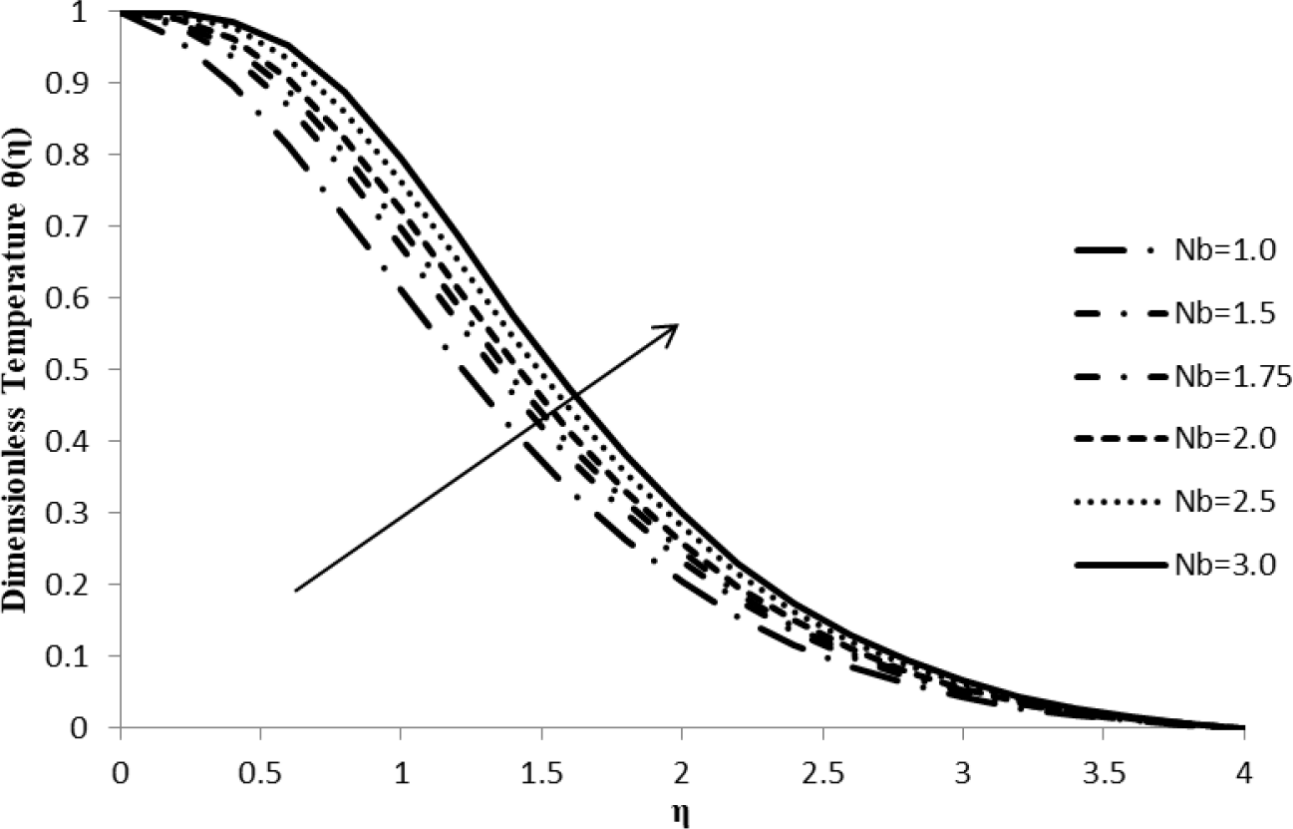
Influence of the Brownian motion parameter Nb on the temperature distribution, θ(η) when *n* = 2.0, ξ = 1.0, *M* = *Q* = 0.0, Pr = 2.0, Nt = 0.5, Le = 5.0, and *F*_w_ = 0.2.

The Brownian motion parameter, Nb, has a remarkable effect on the temperature gradient, θ(η). When Nb increases, it leads to an increase in θ(η) which causes an increase in the thermal boundary layer thickness of the fluid flow. Higher Brownian motion is responsible for fast movement of fluid particles which, in turn, induces either an increase in acceleration or random acceleration levels resulting in additional energy among particles and, consequently, an increase in the temperature gradient and thermal boundary layer thickness (Figure 8).

### Impact of Nb on ϕ(η)

**Figure 9:**
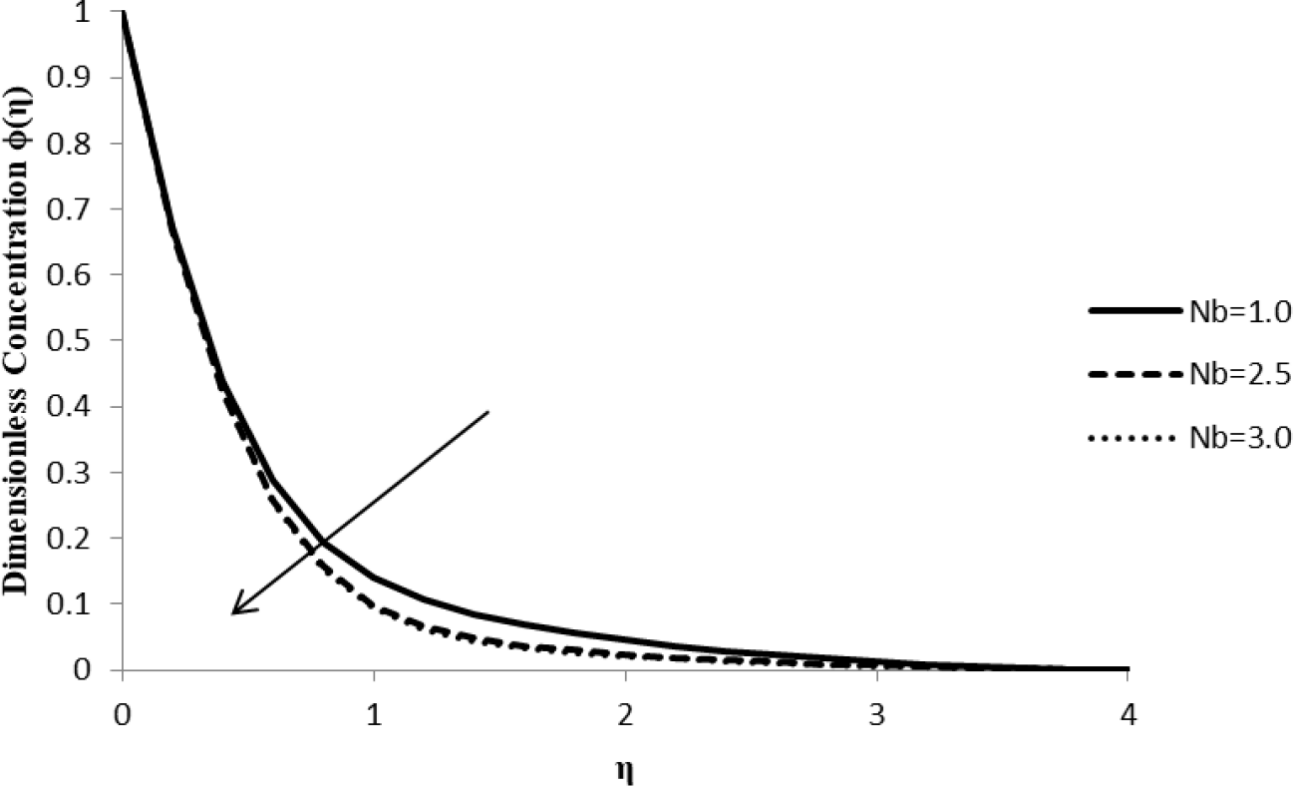
Influence of the Brownian motion parameter Nb on the concentration distribution ϕ(η) when *n* = 2.0, ξ = 1.0, *M* = *Q* = 0.0, Pr = 2.0, Nt = 0.5, Le = 5.0, and *F*_w_ = 0.2.

An increase in the Brownian motion parameter, Nb, results in a decrease in the concentration gradient of the fluid, ϕ(η), due to the fact that the particles move from a high to a low concentration region. As the movement of the fluid particles intensifies with an increase in the Brownian motion, the particles start moving rapidly from regions of higher to regions of lower concentration since the random acceleration decreases the concentration gradient of the fluid flow (Figure 9).

### Impact of Nt on θ(η)

**Figure 10:**
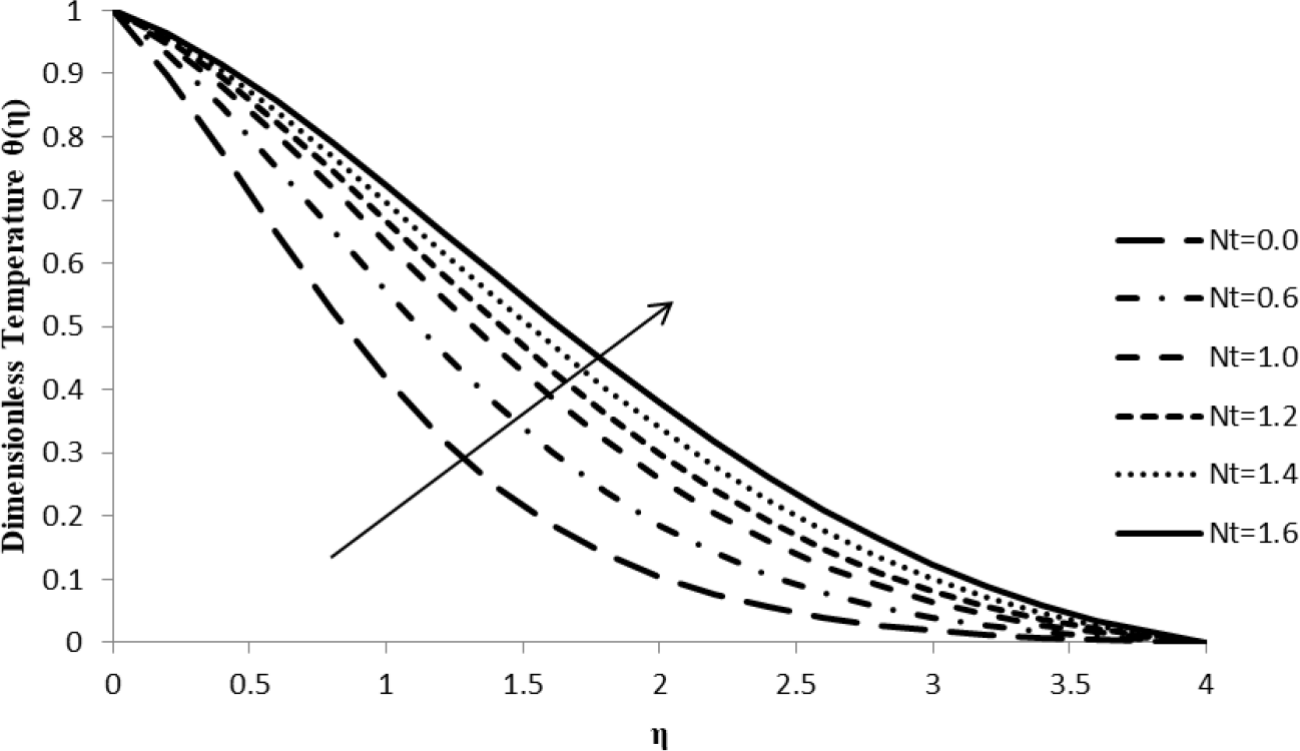
Influence of the thermophoresis parameter Nt on the temperature distribution θ(η) when *n* = 2.0, ξ = 1.0, *M* = *Q* = 0.0, Pr = 2.0, Nb = 0.5, Le = 5.0, and *F*_w_ = 0.2.

With an increase in the thermophoresis parameter, Nt, the temperature gradient of the fluid, θ(η), also increases leading to an enhancement in the thermal boundary layer thickness. Due to the presence of a temperature gradient, different particles of the fluid exhibit different responses to the change in the thermophoresis parameter. As Nt increases, the particles start moving rapidly which causes an elevation in the kinetic energy of the system, resulting in an increase in the temperature distribution and in the boundary layer thickness (Figure 10).

### Impact of Nt on ϕ(η)

**Figure 11:**
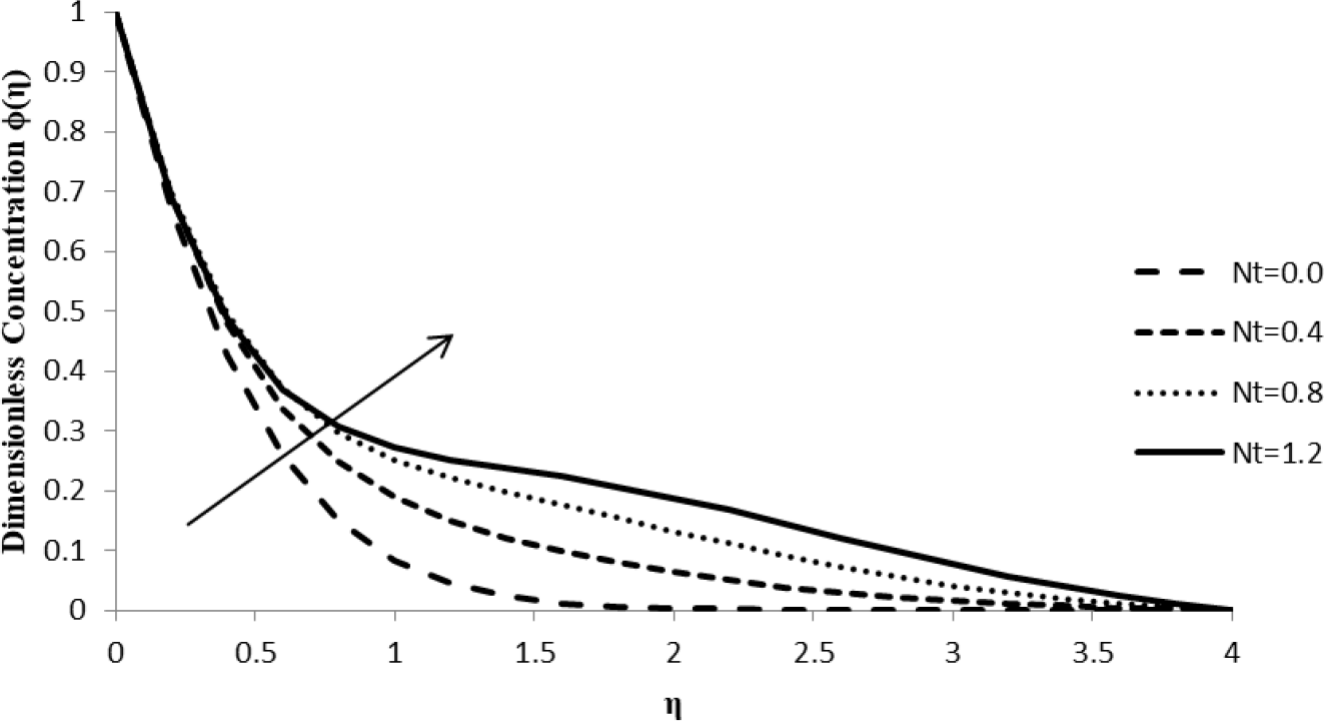
Influence of the thermophoresis parameter Nt on the concentration distribution ϕ(η) when *n* = 2.0, ξ = 1.0, *M* = *Q* = 0.0, Pr = 2.0, Nb = 0.5, Le = 5.0, and *F*_w_ = 0.2.

A small increase in thermophoresis parameter, Nt, causes a massive increase in the concentration distribution of the fluid flow, ϕ(η), which converges to zero at the boundary layer. A small change in the thermophoresis parameter leads to rapid motion in the fluid particles creating excess heat energy and leading to a massive increase in the concentration distribution. Therefore, Figure 11 shows a significant increase in the concentration distribution with a very slight increase in the value of Nt.

### Impact of *M* on *f*´(η)

**Figure 12:**
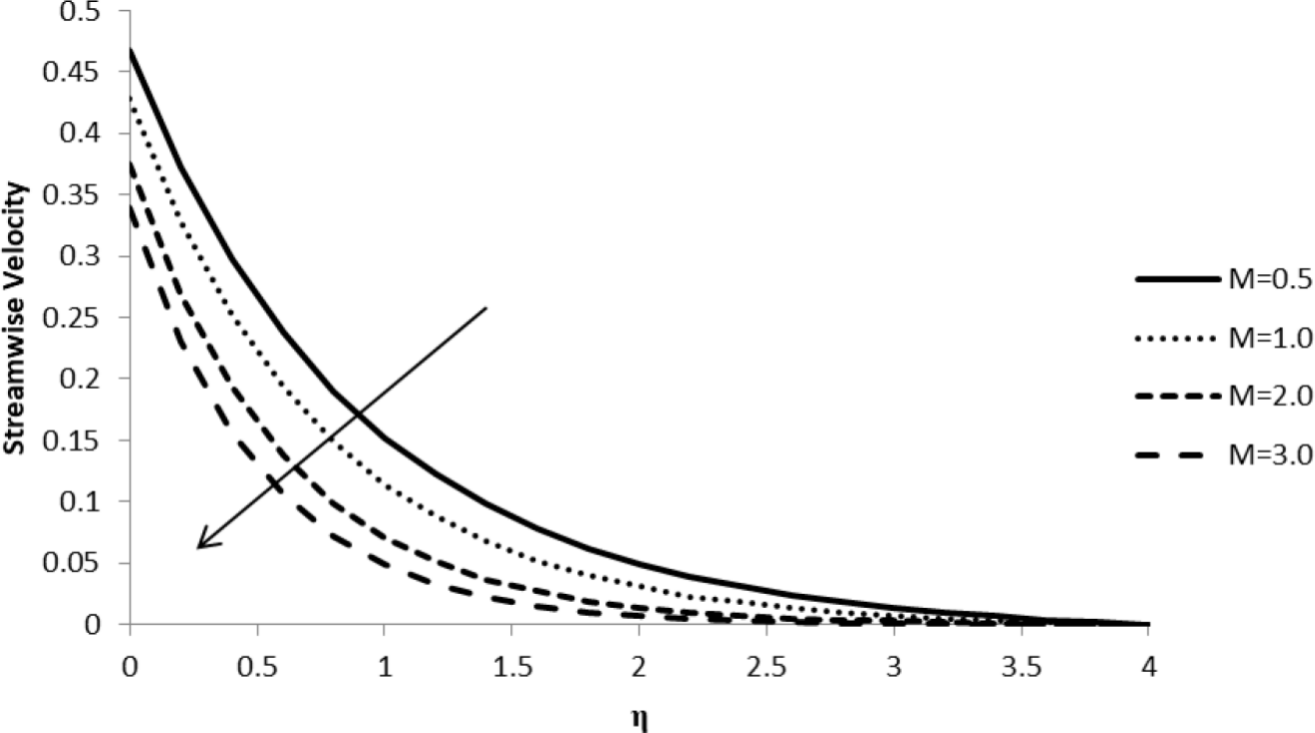
Influence of the magnetic parameter *M* on the streamwise velocity component *f*´(η) when *n* = 2.0, Pr = 2.0, Nb = Nt = 0.5, *Q* = 0.0, Le = 5.0, ξ = 1.0, and *F*_w_ = 0.2.

An increase in the magnetic parameter, *M*, leads to a decrease in the streamwise velocity component, *f*´(η), which, in turn, reduces the velocity boundary layer thickness. The external magnetic field has a massive effect on the velocity profile of an electrically conducting fluid, which causes a considerable amount of resistance to its motion and a driving force, called the Lorentz force, which reduces the fluid velocity (Figure 12).

### Impact of M on θ(η)

**Figure 13:**
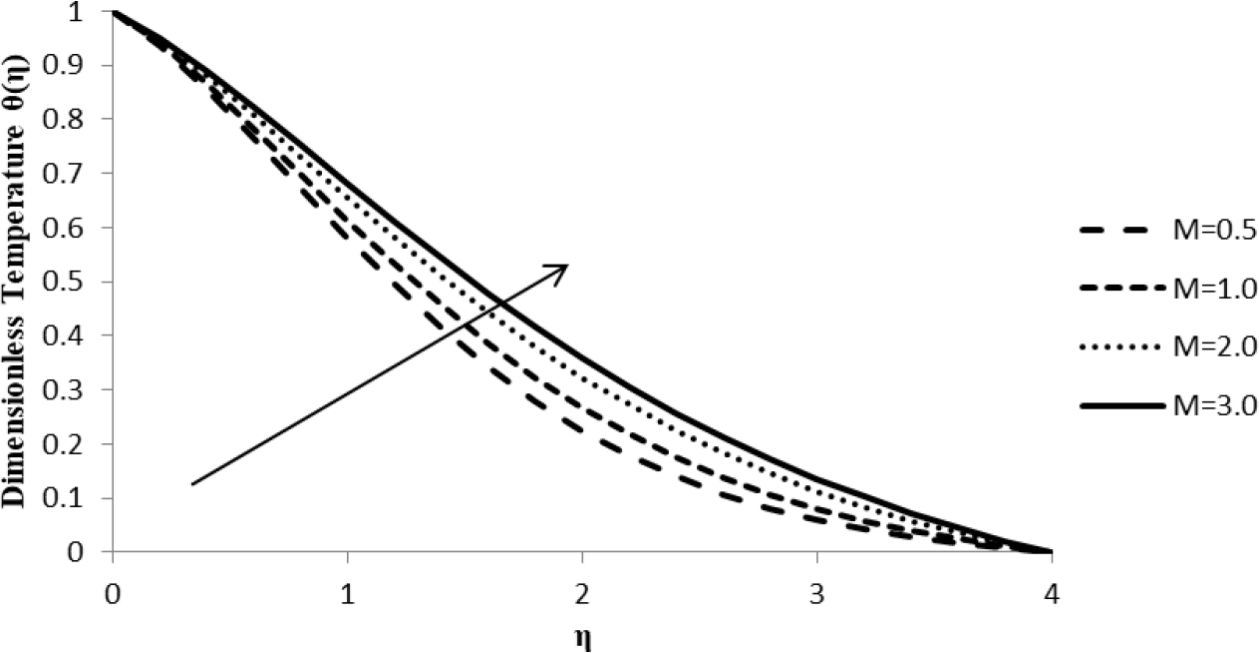
Influence of the magnetic parameter *M* on the temperature distribution θ(η) when *n* = 2.0, Pr = 2.0, Nb = Nt = 0.5, *Q* = 0.0, Le = 5.0, ξ = 1.0, and *F*_w_ = 0.2.

With an increase in the magnetic parameter, *M*, and, consequently, of the fluid resistance, the fluid temperature profile, θ(η), increases and so does the thickness of the thermal boundary layer. This is due to the fact that with an external magnetic field being employed, the temperature of the fluid increases, resulting in the rapid movement of the fluid particles which increases both the thermal energy and the boundary layer thickness and reduces the heat transfer from the sheet (Figure 13).

### Impact of *M* on ϕ(η)

**Figure 14:**
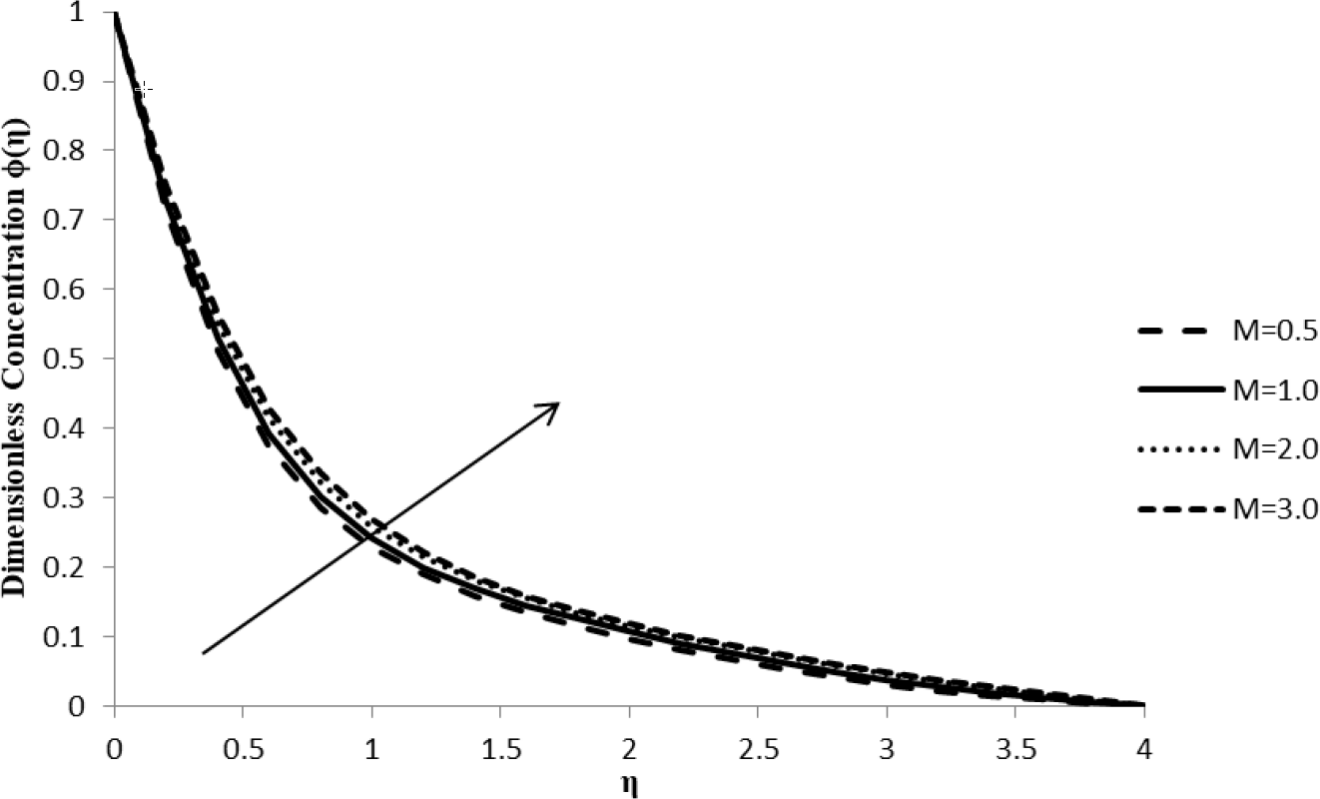
Influence of magnetic parameter *M* on the concentration distribution ϕ(η) when *n* = 2.0, Pr = 2.0, Nb = Nt = 0.5, *Q* = 0.0, Le = 5.0, ξ = 1.0, and *F*_w_ = 0.2.

An increase in the magnetic parameter, *M*, does not have much influence on the concentration distribution, ϕ(η), which is depicted in Figure 14. Even though there is a minimal increasing trend observed, the flow seems invariable. Since the concentration gradient of the fluid flow is not significantly modified by the external magnetic field applied, there is not much of a change in particle motion upon an increase in *M* values (Figure 14).

### Impact of *Q* on *f*´(η)

**Figure 15:**
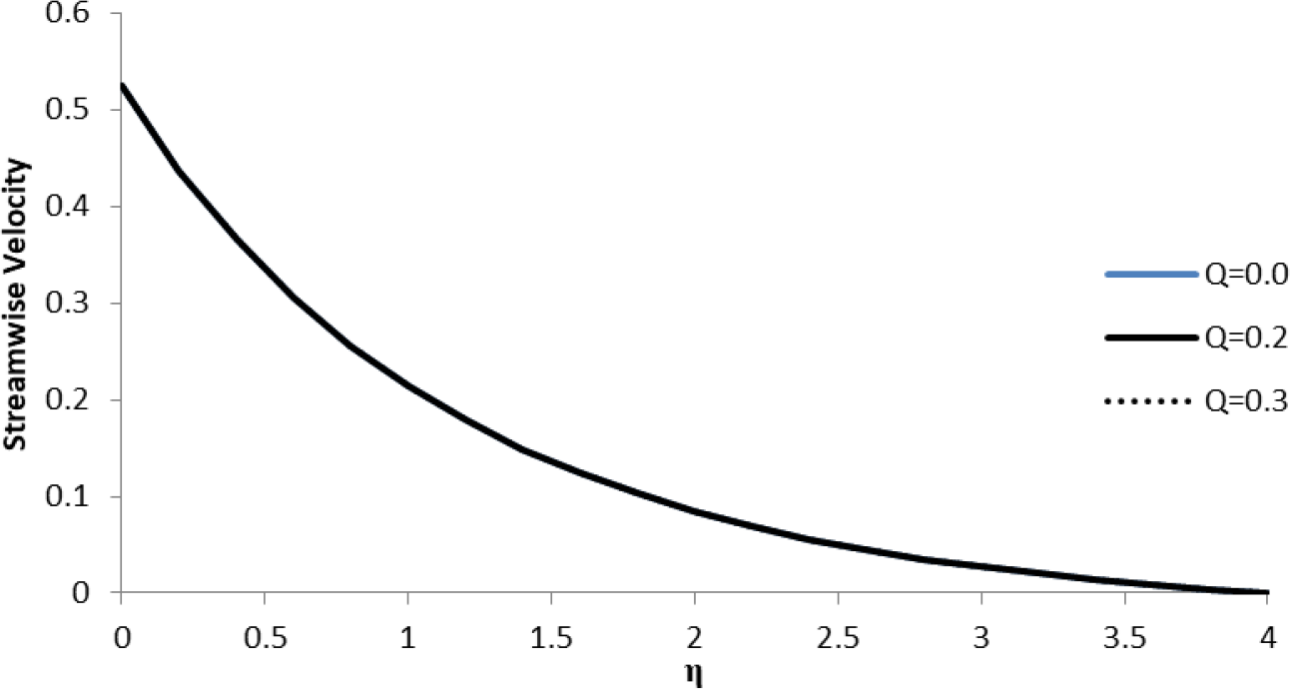
Influence of the heat generation/absorption coefficient *Q* on the streamwise velocity component *f*´(η) when *n* = 2.0, Pr = 2.0, Nb = Nt = 0.5, *M* = 0.0, Le = 5.0, *F*_w_ = 0.2, and ξ = 1.0.

The effect of the heat generation/absorption coefficient, *Q*, on the streamwise velocity component, *f*´(η), is negligible and, therefore, it does not affect the fluid flow velocity. The value of the heat generation/absorption coefficient being positive demonstrates that the heat generated does not have much impact on the velocity gradient of the fluid as the fluid particles move at the same velocity (Figure 15).

### Impact of *Q* on θ(η)

**Figure 16:**
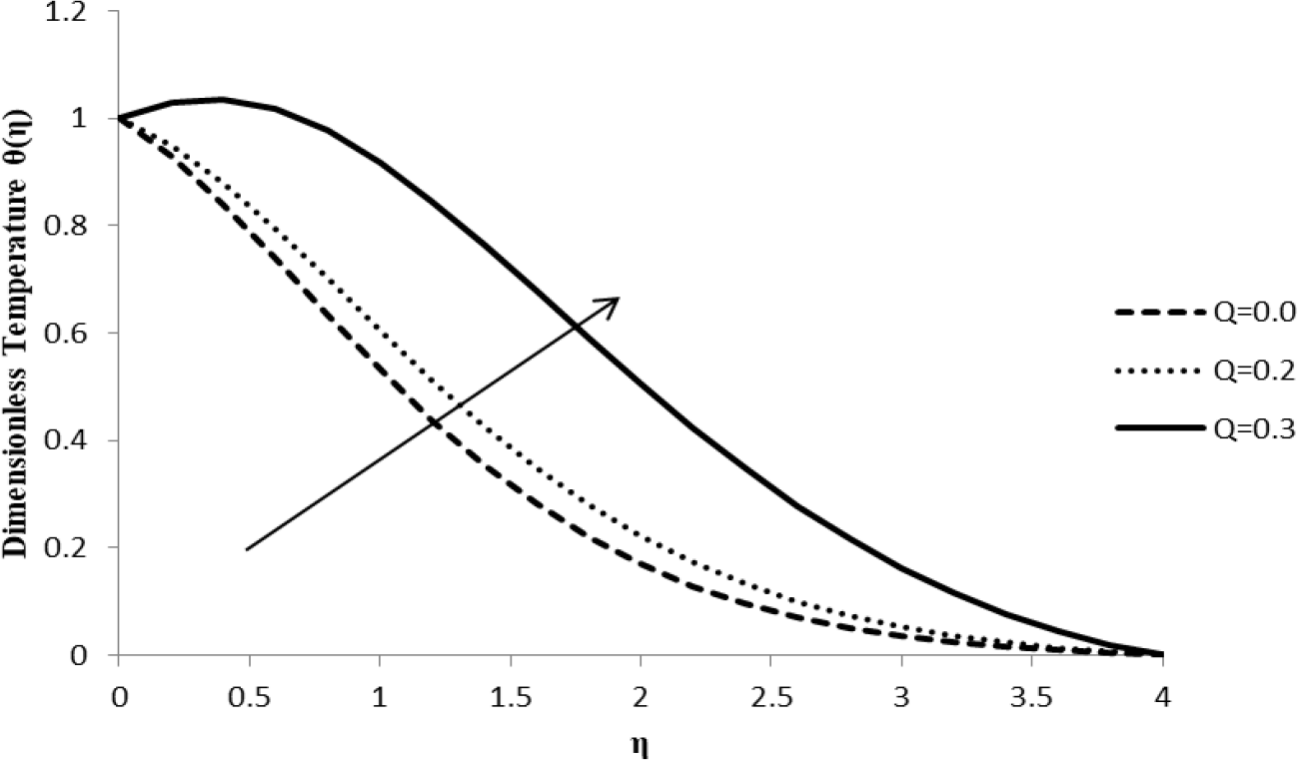
Influence of the heat generation/absorption coefficient *Q* on the temperature distribution θ(η) when *n* = 2.0, Pr = 2.0, Nb = Nt = 0.5, *M* = 0.0, Le = 5.0, *F*_w_ = 0.2, and ξ = 1.0.

The heat generation/absorption coefficient, *Q*, has a considerable effect on the dimensionless temperature profile of the fluid, θ(η), which increases with an increase in the value of *Q* as shown in Figure 16. An increase in the fluid temperature increases the thermal boundary layer thickness. The presence of an external heat source has a significant impact on the temperature gradient of the fluid, resulting in an increase in both the temperature distribution and thermal state of the fluid. With a massive amount of heat energy generated among fluid particles, the thermal boundary layer thickness increases to a larger extent (Figure 16).

### Impact of *Q* on ϕ(η)

**Figure 17:**
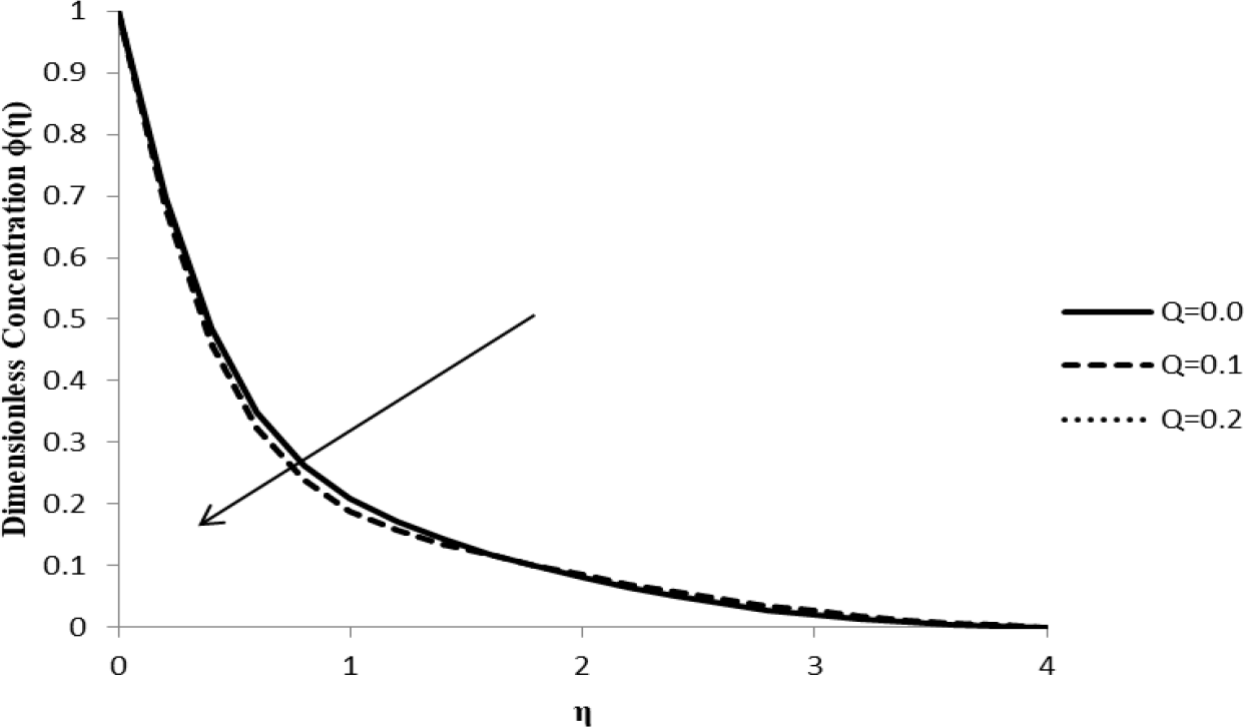
Influence of the heat generation/absorption coefficient *Q* on the concentration distribution ϕ(η) when *n* = 2.0, Pr = 2.0, Nb = Nt = 0.5, *M* = 0.0, Le = 5.0, *F*_w_ = 0.2, and ξ = 1.0.

The heat generation/absorption coefficient, *Q*, does not have much of an effect on the concentration distribution, ϕ(η), as the concentration of the fluid remains unaltered with the change of heat in the fluid. The heat generation coefficient is responsible for changing the heat gradient of the fluid flow, which has a negligible influence on the concentration levels of the fluid particles (Figure 17).

## Conclusion

In this work, it was found that an increase in the slip parameter, ξ, significantly reduces the fluid streamwise velocity gradient which, in turn, reduces the boundary layer thickness. On the other hand, an increase in ξ causes an increase in both the temperature θ(η) and concentration gradients ϕ(η). It was also found that an increase in the stretching parameter *n* reduces the velocity gradient, thus, depleting the boundary layer; however, the temperature and nanoparticle concentration of the flow were found to increase. An increase in the Brownian motion parameter, Nb, was found to increase the temperature gradient but reduce the concentration of the fluid, whereas the increase in the thermophoresis parameter, Nt, was found to increase both θ(η) and ϕ(η). The external magnetic field was found to have an enormous influence on the velocity gradient of the fluid which decreases with an increase in *M* due to a drag-like force, or resistance, that is developed by the fluid. Another observation was that the temperature gradient considerably increases with an increase in *M*, thus enhancing the thermal boundary layer thickness of the fluid as the external magnetic field does not have much of an effect on the concentration gradient. It was also shown that an increase in the heat generation/absorption coefficient, *Q*, results in a significant increase in the temperature profile without changing the velocity or the concentration of the fluid. With *Q* being positive, there is heat generation across the flow, which enhances the thermal boundary layer thickness.

## References

[1] Sakiadis B C (1961). AIChE J.

[2] Sakiadis B C (1961). AIChE J.

[3] Crane L J (1970). Z Angew Math Phys.

[4] Chamhka A J, Issa C (2000). Int J Numer Methods Heat Fluid Flow.

[5] Anderson J D (2009). Explicit Finite Difference Methods: Some Selected Applications to Inviscid and Viscous Flows. Computational Fluid Dynamics.

[6] Khan W A, Pop I (2010). Int J Heat Mass Transfer.

[7] Rana P, Bhargava R (2012). Commun Nonlinear Sci Numer Simul.

[8] Das K (2015). J Egypt Math Soc.

[9] Hayat T, Imtiaz M, Alsaedi A (2015). Appl Math Mech (Engl Ed).

[10] Besthapu P, Bandari S (2015). J Appl Math Phys.

[11] Abdul Gaffar S, Ramachandra Prasad V, Keshava Reddy E, Anwar Bég O (2015). Ain Shams Eng J.

[12] Dogonchi A S, Ganji D D (2016). J Mol Liq.

[13] Farooq M, Anjum A, Hayat T, Alsaedi A (2016). J Mol Liq.

[14] Qayyum S, Hayat T, Alsaedi A (2017). Results Phys.

[15] Sreekala B, Janardhan K, Ramya D, Shravani I (2017). Global J Pure Appl Math.

[16] Rashid I, Ul Haq R, Al-Mdallal Q M (2017). Phys E (Amsterdam, Neth).

[17] Ahmad R, Mustafa M, Turkyilmazoglu M (2017). Int J Heat Mass Transfer.

[18] Seth G S, Bhattacharyya A, Kumar R, Chamkha A J (2018). Phys Fluids.

[19] Soomro F A, Ul Haq R, Al-Mdallal Q M, Zhang Q (2018). Results Phys.

[20] Soomro F A, Usman M, Ul Haq R, Wang W (2018). Int J Heat Mass Transfer.

[21] Farooq A, Ali R, Benim A C (2018). Phys A (Amsterdam, Neth).

[22] Irfan M, Farooq M A, Iqra T (2019). Front Phys.

[23] Pal D, Mondal S, Mondal H (2019). Int J Ambient Energy.

[24] Shah Z, Babazadeh H, Kumam P, Shafee A, Thounthong P (2019). Front Phys.

[25] Yousif M A, Ismael H F, Abbas T, Ellahi R (2019). Heat Transfer Res.

